# West Nile Virus Drug Discovery

**DOI:** 10.3390/v5122977

**Published:** 2013-12-03

**Authors:** Siew Pheng Lim, Pei-Yong Shi

**Affiliations:** Novartis Institute for Tropical Diseases, 10 Biopolis Road, Chromos 05-01, Singapore 138670, Singapore; E-Mail: siew_pheng.lim@novartis.com

**Keywords:** drug discovery, antiviral, West Nile virus, flavivirus

## Abstract

The outbreak of West Nile virus (WNV) in 1999 in the USA, and its continued spread throughout the Americas, parts of Europe, the Middle East and Africa, underscored the need for WNV antiviral development. Here, we review the current status of WNV drug discovery. A number of approaches have been used to search for inhibitors of WNV, including viral infection-based screening, enzyme-based screening, structure-based virtual screening, structure-based rationale design, and antibody-based therapy. These efforts have yielded inhibitors of viral or cellular factors that are critical for viral replication. For small molecule inhibitors, no promising preclinical candidate has been developed; most of the inhibitors could not even be advanced to the stage of hit-to-lead optimization due to their poor drug-like properties. However, several inhibitors developed for related members of the family *Flaviviridae*, such as dengue virus and hepatitis C virus, exhibited cross-inhibition of WNV, suggesting the possibility to re-purpose these antivirals for WNV treatment. Most promisingly, therapeutic antibodies have shown excellent efficacy in mouse model; one of such antibodies has been advanced into clinical trial. The knowledge accumulated during the past fifteen years has provided better rationale for the ongoing WNV and other flavivirus antiviral development.

## 1. Introduction

West Nile virus (WNV) has an enveloped virion of about 50 nm in diameter, and comprises a lipid bilayer that surrounds a nucleocapsid with a single-stranded, positive-sense RNA genome of approximately 11,000 nucleotides. Both the 5’ and 3’ noncoding regions of the genome form extensive secondary structures, which are important for translation, RNA synthesis, and packaging [1,2,3]. The viral RNA is translated as a single polyprotein that is post- and co-translationally cleaved by both host and viral proteases to form three structural (capsid, envelope, and pre-membrane) and seven nonstructural (NS1, NS2A, NS2B, NS3, NS4A, NS4B, and NS5) proteins [4]. The envelope (E) protein is involved in receptor interaction, membrane fusion, and virion assembly. The pre-membrane (prM) stabilizes the conformation of E during virion assembly and protects E from undergoing premature fusion during virus exocytosis to the cell surface. The capsid (C) protein encapsidates the viral genome during assembly. Viral replication and assembly takes place in the cytoplasm, with budding in the endoplasmic reticulum (ER). The nonstructural proteins together form the replication complex needed for viral RNA synthesis and virion formation. NS1 glycoprotein is anchored to the cell surface and also secreted. It plays a role in replication although its exact role is not fully determined [5]. There is also evidence that NS1 is involved in neuroinvasiveness of WNV [6]. NS3 is a multi-functional protein that has protease, helicase, and NTPase activities. NS3 protease acts together with NS2B, is responsible for cleaving other nonstructural proteins from the viral polyprotein. NS5 protein encodes the viral methyltransferase (MTase) and RNA-dependent RNA polymerase (RdRp). NS4A induces membrane rearrangements, which are important for formation of the viral replication complex [7]. Several of the nonstructural proteins, including NS2A, NS2B, NS4A, and NS4B, are transmembrane proteins that have no identified enzymatic activities, but are essential for formation of the active replication complex [8,9]. They have also been shown to inhibit one or more components of the innate immune response against viral infection [10]. 

There are currently no marketed drugs or clinical candidates for treatment or prevention of flavivirus (including WNV) infection in humans. This article reviews the latest development in WNV drug discovery and the challenges/opportunities ahead.

## 2. Approaches to Identify Antiviral Inhibitors

Effective antiviral therapy for WNV may target either viral (structural and non-structural) or host proteins that are essential for WNV infection or replication. Small molecule-based inhibitors can be identified using the following approaches. (i) HTS (high-throughput screening) using virus replication assays; (ii) HTS using viral enzyme assays; (iii) structure-guided *in silico* docking and rational design; (iv) Repurposing other viral inhibitors for WNV. It is envisaged that inhibitory compounds that act on related flaviviruses, such as dengue virus (DENV) or hepatitis C virus (HCV) may also be effective on WNV. There are some examples with pan-active flavivirus entry, protease, MTase, and RdRp (nucleoside) inhibitors. Many of the drug screening and design efforts against WNV have thus far centered on the viral protease, with fewer endeavors directed at the other viral enzymatic activities, such as the RdRp or MTase. Non-structural proteins with no enzymatic activities (NS2A, NS4A, and NS4B) could also be targeted for antiviral development, as demonstrated by the success of HCV NS5A inhibitor currently in clinical trial [11]. 

Besides small molecule-based inhibitors, therapeutic antibodies have been vigorously pursued for WNV treatment. So far, therapeutic antibodies represent the most promising approach. This approach has not only produced candidates in clinical trial for treatment of WNV infection, but also helped to understand antibodies that are needed for an effective flavivirus vaccine. 

## 3. Inhibitors of Viral Targets

### 3.1. Viral Entry Inhibitors

Entry inhibitors prevent virus from attachment to cell, entering into cell, or virus-host membrane fusion. Flavivirus E protein’s major conformational changes and well-defined molecular structures, both pre- and post-fusion, are potentially amenable to inhibitor design [12,13,14]. In particular, the crystal structure of the DENV2 E protein displays a ligand-binding pocket that was occupied by a detergent molecule, n-octyl-b-D-glucoside (b-OG) [15]. This initiated several groups to identify and optimize potential inhibitors targeting this region of E protein for DENV [16,17,18,19,20] and YFV [21,22,23], mainly through a virtual screening approach. Although different classes of compounds were identified that inhibited DENV, only a handful worked on WNV. One compound (compound 5) was reported to exhibit anti-DENV2 and WNV activities with EC_50_ values of 1.2 ± 0.7 and 3.8 ± 2.9 µM respectively [20]. From a virtual screening campaign, another compound (compound 1), belonging to the quinazoline scaffold, demonstrated a broad spectrum anti-flavivirus activity [19]; further optimization resulted in compound 6 with submicromolar activities against both DENV1-4 and WNV. Despite efforts to improve the pharmacokinetic properties, its low solubility prevented further development.

Besides small molecule inhibitors, protein- and peptide-based inhibitors have also been pursued to inhibit WNV entry. One group demonstrated that recombinant domain III from WNV E protein inhibited WNV entry into Vero cells and C6/36 mosquito cells [24]. Short peptides (25–33 aa) derived from DENV and WNV envelope protein sequences can inhibit DENV2 and WNV infection in cell culture with EC_50_ of about 10 µM [25]. The drawback of peptidic inhibitors is the need for intravenous administration and its limited shelf life, which limits their use in clinical settings, especially in developing countries.

### 3.2. Therapeutic Antibody

The therapeutic antibody represents the most promising class of WNV entry inhibitors among all the current antiviral approaches. Antibodies can protect against flavivirus infection through several mechanisms, including blockage of receptor binding, inhibition of viral fusion, Fc-γ receptor-dependent viral clearance, complement-mediated lysis of virus or infected cells, and antibody-dependent cytotoxicity of infected cells. Readers are encouraged to read an excellent recent review on this topic [26]. Small numbers of clinical studies showed that patients with neuroinvasive WNV infection improved after receiving immune γ-globulin from Israeli donors who were serum-positive against WNV [27,28,29,30]. These clinical results encourage the development of human or humanized monoclonal antibodies for treatment of WNV infection. Indeed, potent monoclonal antibody fragments have been developed against WNV. Mice and hamsters infected with WNV were protected after a single-dose treatment of such antibody on day 5 or 6 post infection [31,32,33]. A phase I clinical trial completed in 2009, determined that a potent antibody (derived from E16 antibody, also known as MGAWN1 was safe and well tolerated in healthy subjects as single infusions up to 30 mg/kg. Unfortunately, a phase II trial to assess its efficacy in severe WNV infection in humans was terminated prematurely due to poor patient enrollment. 

### 3.3. NS3

**Figure 1:**
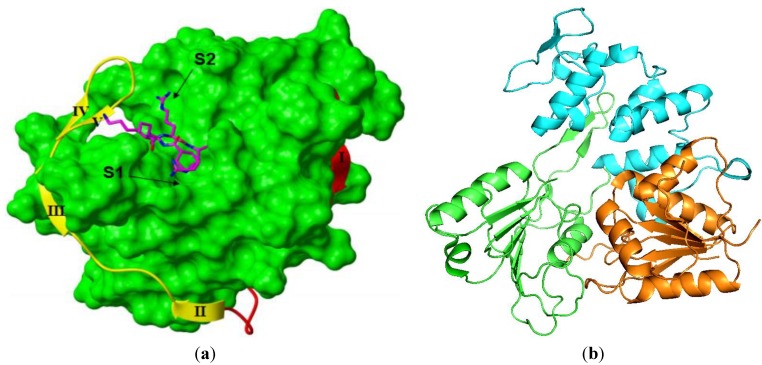
Crystal structures of West Nile Virus (WNV) NS2/NS3 protease and NS3 helicase domains depicted in cartoon representation. (**a**) WNV NS2B/3 protease bound to the peptidic inhibtor, nKRR-H inhibitor [PDB code 2FP7; 39]. NS3 is colored green. NS2B is shown in red (*N*-terminal region) and yellow (*C*-terminal region). Bz-nKRR-H (pink) is shown in stick representation. Locations of S1 and S2 pockets are marked with arrows; (**b**) WNV NS3 helicase with domains 1, 2 and 3 colored in cyan, green and orange, respectively [PDB code 2QEQ; 40].

NS3 is a multifunctional protein, consisting of the *N*-terminal serine protease domain localized to amino acids 1–169 and the *C*-terminal domain from residues 180–618, bearing helicase, nucleoside triphosphatase, and RNA triphosphatase activities [34,35,36]. The *N* and *C*-terminal domains are linked via a flexible inter-domain, comprising residues 169–179 of NS3 [37,38]. Along with other flaviviruses, the crystal structures of the WNV protease and helicase domains have been resolved ([39,40,41,42,43]; Figure 1). Full-length NS3 has increased ATP binding and helicase activity compared to the helicase domain alone [40], but no enhancement of the protease activity compared to the protease domain alone [37]. One group found that the functional NS2B/3 protease did not influence helicase activity [40], whilst another group found that it represses helicase unwinding activity [44]. Both the ATPase and helicase activities of NS3 have been shown to be regulated by NS4A [45], and the two activities can function independently of each other [46]. Within infected host cells, these functions appear to be regulated by their differential localization to separate virus-induced membranous compartments [47].

#### 3.3.1. NS3 Protease

WNV NS3 protease, like the counterpart from other members of the flaviviruses, is activated by its membrane associated co-factor, the NS2B protein [48,49]. Besides cleaving the junctions between the viral proteins, cleavage of host proteins by WNV NS2B/3pro has been proposed to contribute to neuro-pathogenesis [50,51,52]. Therapeutic strategies for viral proteases have been successfully exemplified by ten HIV-1 protease inhibitors (PIs; [53]) and two recent HCV protease inhibitors [54]. Thus, protease inhibitors against flaviviruses would also likely be efficacious in the clinic and have been intensely pursued. Nevertheless, in both HIV-1 and HCV patients, rapid emergence of PI drug-resistant viruses due to expansion of pre-existing naturally resistant variants [55,56,57] have been observed and is the reason to be cautious about this approach. Although WNV disease is primarily acute in nature, chronic and persistent infections in humans [58] with serious long-term sequelae have also been reported [59,60,61]. Fortunately, sequence similarity across WNV lineages 1 and 2 for NS3 protease region is greater than 96%, implying that genetic barrier to any naturally occurring mutant WNV variants would be higher than for HCV or HIV-1 where the protease sequences are much less conserved. 

**Table 1:** List of WNV NS2B/3pro peptidic inhibitors.

Compound	WNV Ki (µM)	Binding Mode	Co-crystallised with WNV NS2B/3pro	Anti-WNV cellular activity	Ref
Aprotinin	0.026; 0.09 ± 0.02	non-covalent	34		[73,74]
Bz-Nle-KRR-H	4.1	covalent; warhead	31		[70]
Bz-Nle-KKR-H	1.9	covalent; warhead			[70]
Bz-Nle-KR(p-guanidinyl)F-H	12	covalent; warhead			[70]
rrrrrr-NH2 (hexa-D-R-NH2)	0.478	non-covalent			[75]
rrrrrrr-NH2 (hepta-D-R-NH2)	0.041	non-covalent			[75]
rrrrrrrr-NH2 (octa-D-R-NH2)	0.017	non-covalent			[75]
rrrrrrrrr-NH2 (nona-D-R-NH2)	0.006	non-covalent		yes	[75]
rrrrrrrrrr-NH2 (deca-D-R-NH2)	0.002	non-covalent			[75]
rrrrrrrrrrr-NH2 (undeca-D-R-NH2)	0.001	non-covalent			[75]
rrrrrrrrrrrr-NH2 (dodeca-D-R-NH2)	0.001	non-covalent			[75]
2-naphthoyl-KKR-H	0.041	covalent; warhead	33		[71]
phenylacetyl-KKR-H	0.009; 0.70 ± 0.04	covalent; warhead		yes	[71,72]
4-phenylphenylacetyl-KKR-H	0.006; 0.056 ± 0.004	covalent; warhead			[71,72]
acetyl-KKR-H	0.49 ± 0.32	covalent; warhead			[72]
propionyl-KKR-H	0.43 ± 0.06	covalent; warhead			[72]
cyclopropionyl-KKR-H	0.19 ± 0.01	covalent; warhead			[72]
benzoyl-KKR-H	0.21 ± 0.09	covalent; warhead			[72]
acetyl-KR-H	0.09 ± 0.02	covalent; warhead			[76]
propionyl-KR-H	0.17 ± 0.06	covalent; warhead			[76]
cyclopropionyl-KR-H	0.22 ± 0.05	covalent; warhead			[76]
benzoyl-KR-H	0.92 ± 0.09	covalent; warhead			[76]
acetyl-Lys-Lys-agmatine	9.1 ± 2.1	non-covalent			[77]
4-phenylphenylacetyl-Lys-Lys-agmatine	2.05 ± 0.13	non-covalent			[77]
2-chloro-4-phenyl-phenacetyl-L-Lys-Lys-agmatine	1.3 ± 0.2	non-covalent			[78]
4-chloro-4-phenyl-phenacetyl-L-Lys-Lys-agmatine	2.4 ± 0.5	non-covalent			[78]
2-methyl-4-phenyl-phenacetyl-L-Lys-Lys-agmatine	3.4 ± 0.6	non-covalent			[78]
4-methyl-4-phenyl-phenacetyl-L-Lys-Lys-agmatine4	3.5 ± 0.7	non-covalent			[78]

Due to the large amount of structural, biochemical, and functional information garnered on this enzyme over the past decade (refer to [62,63,64,65] for excellent reviews on these topics), many researchers have taken either a rational design approach to find inhibitors via substrate mimicry or utilized *in silico* docking methodologies (Table 1). The challenges with the peptidomimetics approach for WNV protease are the shallow active site and the apparent flexibility of NS2B residues that contribute to the active site, as shown by the apo- and inhibitor-bound crystal structures ([39,41,42,43,66]; Figure 1) as well as NMR structures of this enzyme [67,68,69]. There is also a need to replace the two conserved basic P1 and P2 residues of the substrate cleavage site (P1-Arg and P2-Lys). Thus far, no group has demonstrated any success in this area. In general, researchers have been able to design more potent peptidic inhibitors against WNV protease compared to DENV protease. Single or double digit nano-molar inhibitors have been reported for the former, whilst none has been found for DENV protease. 

As observed by earlier reports, selectivity among DENV, YFV, and WNV proteases may be achieved through the P2 site whereby the WNV enzyme prefers Lys over Arg. Substituents that increase the bulkiness of the P2-Lys group are also not accommodated [70,71,72]. Two highly potent peptidic inhibitors were reported to have cellular anti-WNV activity ([71,75]; Table 1). However, the physiochemical and pharmacokinetic properties of both nona-D-R-NH2 and phenylacetyl-KKR-H make them unsuitable drug candidates as they are highly charged (presence of large numbers of Arg residues), lack specificity (aldehyde warhead is highly reactive and nonspecific), and would also be rapidly degraded in plasma. Interestingly, di-peptidic aldehyde inhibitors with small caps exhibit improved potencies compared to their tripeptide counterparts ([76]; Table 1). One of the most potent inhibitor is a simple di-peptide, acetyl-KR-H, with Ki of 90 nM [76]. This inhibitor may be a good starting point for peptidomimetics. Recently, several non-covalent peptide inhibitors with P1 decarboxylated arginine (agmatine; 4-aminobutylguanidine) were designed and tested against WNV protease ([77,78]; Table 1]. Interestingly, for this class of inhibitors, di-peptide with bulky aromatic caps are more potent than those with small caps, which suggest that in the absence of a warhead, interactions beyond S1 and S2 subsites contribute more significantly to the binding affinity. Whilst promising, this class of inhibitor still retains the highly charged nature of the covalent peptidic inhibitors. 

Nitsche *et al*. [79] reported the generation of 3-aryl-2-cyanoacrylamide compounds, which are not based on substrate mimetics. These compounds exhibited double-digit micromolar activities against WNV and DENV proteases; their binding specificity was demonstrated using a competition assay with aprotinin. The advantages of these compounds are the high ligand efficiency and the possibility to “grow” them to improve their potency.

One final consideration for development of WNV protease inhibitors is the need to possess selectivity over other arginine-specific host proteases such as trypsin, thrombin, factor Xa, and furin. Nevertheless, much progress has been made to generate selective inhibitors to these various human enzymes. Dabigatran is a highly potent inhibitor of thrombin that was generated through structure guided design [80], whilst Rivaroxaban is a nonpeptide inhibitor against FactorXa that was identified from a screening campaign [81]. With perseverance, it would be a matter of time before success can be achieved too in WNV protease inhibitors.

**Table 2:** List of WNV NS2B/3pro non-peptidic inhibitors.

Compound (Substrate)	Core	Method	WNV IC_50_ (µM)	Biophysical method (*K*_d_, µM)	Anti-WNV cellular activity EC_50_ [CC_50_] in µM	Ref
cpd A (Boc-GKR-AMC)	8-hydroxyquinoline	Diverse library screening	6.4 ± 0.6			[82]
cpd B (Boc-GKR-AMC)	8-hydroxyquinoline	Diverse library screening	6.8 ± 1.2; 3.6 ± 2.0		1.4 ± 0.4 [140 ± 1.98]	[82,83]
Compound 14	8-hydroxyquinoline	Derivatization of cpd B	1.0 ± 0.08 (GKR-AMC); 2.01 ± 0.08 (nkRR-AMC)			[83]
Compound 12j (Boc-GKR-AMC)	1-oxo-1,2-dihydroisoquinoline	Focus library design and screening	30			[84]
Palmatine (pERTKR-AMC)	natural product	unknown	96		3.6 [1031]	[85]
cpd 1 (nKRR-AMC)	Carbamimidoylsulfanyl-methyl	*In silico* FBS	178	NMR (40)		[86]
cpd 1 (nKRR-AMC)		*In silico* FBS	2.8 ± 0.1	NMR (90 ± 40)		[87]
cpd 1 (nKRR-AMC)		*In silico* FBS	34.2 ± 0.1			[87]
SID-852843 (Pyr-RTKR-AMC)	pyrazolyl benzoic acid ester	Diverse library screening	0.105			[88]
SID-4245669 (Pyr-RTKR-AMC)	pyrazolyl benzoic acid ester	Diverse library screening	0.11			[88]
SID-3717586 (Pyr-RTKR-AMC)	pyrazolyl benzoic acid ester	Diverse library screening	1.353			[88]
cpd 7a		Derivatization of pyrazole ester	1.96			[89]
cpd 10a		Derivatization of pyrazole ester	4.03			[89]
cpd 4; 166347 (FRET)	guanidinylated 2,5-dideoxystreptamine	Diverse library screening	1.2 ± 0.3			[74]
cpd 9; 166550 (FRET)	guanidinylated 2,5-dideoxystreptamine	Diverse library screening	4 ± 2			[74]
cpd 7; 166346 (FRET)	guanidinylated 2,5-dideoxystreptamine	Diverse library screening	6 ± 1			[74]
cpd 2; 166750 (FRET)	guanidinylated 2,5-dideoxystreptamine	Diverse library screening	8 ± 1			[74]
cpd 6; 166631 (FRET)	guanidinylated 2,5-dideoxystreptamine	Diverse library screening	8 ± 1			[74]
cpd 1a24 (Pyr-RTKR-AMC)	2-{6-[2-(5-phenyl-4H-[1,2,4]triazol-3-ylsulfanyl)acetylamino]-2-{6-[2-(5-phenyl-4H-[1,2,4]triazol-3-ylsulfanyl)acetylamino]-benzothiazol-2-ylsulfanyl}acetamide	Diverse library screening	3.4 ± 0.2			[90]
cpd 1a16 (Pyr-RTKR-AMC)	1,3,4,5-tetrasubstituted 1H-pyrrol-2(5H)-one	Diverse library screening	2.2 ± 0.7			[91]
cpd 1a40 (Pyr-RTKR-AMC)	9,10-dihydro-3H,4aH-1,3,9,10a-tetraazaphenanthren-4-one	Diverse library screening	2.2 ± 0.7			[92]
Tyrothricin (M23)	decapolypeptide antibiotic	Diverse library screening	2 ± 0.2			[93]
Cpd 1; NSC86314 (Pyr-RTKR-AMC)		*In silico* docking	0.26		42.77 [212.5]	[94]
Cpd 2; NSC16898 (Pyr-RTKR-AMC)		*In silico* docking	0.44		17.01 [235.8]	[94]

HTS using *in vitro* WNV protease biochemical assays as well as *in silico* docking of compounds into the WNV protease structure have also been actively pursued (Table 2). The advantage of these methods is the potential to find allosteric inhibitors that block NS2B/3 interaction or bind outside of the NS3 active site. A number of inhibitors have been identified, but majority of them show micromolar activities in the enzyme assays without biophysical confirmation of their binding specificity. Even fewer demonstrated anti-WNV activity in cell-based assays. This raises the possibility that some of these hits may be non-specific. The exceptions are fragments identified by *in silico* docking which were shown to bind to the protease by NMR [86,87]. Two highly potent classes of compounds were reported to have submicromolar inhibitory activities in the *in vitro* biochemical assays [88,94], with selectivity over DENV2 protease and furin, as well as anti-WNV cellular activity [94]. However, the EC_50_ value has not been shown to be a consequence of on-target inhibition inside the cells. One way to confirm this is to raise resistant WNV against the inhibitors. Unfortunately, none of the published inhibitors have been reported to progress beyond the hit optimization phase. This is probably in part due to the difficulties in obtaining co-crystal structures of these inhibitors bound to the WNV protease, probably due to the flexible NS2B. Devising means for generation of robust co-crystals will pave the way forward for identification of WNV inhibitors. Although many researchers docked the hits into the enzyme active site to assess their binding modes and design analogs for SAR studies, this approach has not proven to be fruitful. 

#### 3.3.2. NS3 Helicase

The *C*-terminal of flavivirus NS3 encompasses helicase, NTPase, and RTPase activities. Adaptive amino acid changes in the WNV helicase have been implicated in virus transmissibility and pathogenesis. A substitution of amino acid T249P in the NS3 helicase (found in North American WNV) in a low-virulence strain was sufficient to generate a phenotype highly virulent to American crows [95]. Furthermore, a virus strain with a S365G mutation in the helicase domain was shown to overcome the host interferon response. This mutation modulated the ATPase activity of NS3 and enabled it to subvert Oas1b-mediated suppression of viral RNA accumulation [96].

Much more efforts had been made to identify inhibitors to WNV helicase compared to efforts for other flavivirus helicase. Nevertheless, similar to the disappointing experiences for WNV protease, WNV inhibitors have also not progressed beyond the hit-to-lead finding phase. In many cases, there was also no report of anti-WNV activity of these compounds in cell culture. An in silico screening campaign using the WNV (Kunjin) helicase found that ivermectin potently inhibited the dsRNA unwinding activity of WNV helicase with IC_50_ values between 200–400 nM [97]. No inhibition of NS3 ATPase or NS5 RdRp activity was observed. Production of infectious WNV was also affected (EC_50_ = 4 μM) and time of addition experiments suggest ivermectin works during viral replication. Ivermectin has been used extensively for more than twenty years, as a broad-spectrum, oral drug against parasitic infections [98]. More recently, it was also shown to inhibit DENV1-4 replication, most likely through interfering with NS5 nuclear translocation [99,100]. Whilst interesting, more work needs to be done to further ascertain the efficacy of ivermectin against flavivirus-associated diseases, such as *in vivo* testing in relevant small animal models, confirmation of direct effect(s) on viral replication, and generation of ivermectin-resistant viruses. Nevertheless, evaluation of antiviral activity of already approved drugs such as ivermectin could streamline pathways to clinical evaluation compared to development of entirely new compounds (See also Section 4 on cyclophilins and Celgosivir).

By randomly screening peptides derived from the helicase protein, Browoski *et al*. [101] found that a basic peptide comprising motif VI (amino acids 1487–1500) of HCV helicase inhibited the unwinding activity of HCV, WNV, and JEV helicases, without affecting their NTPase activity. Interestingly, WNV helicase is more strongly inhibited by the HCV motif VI peptide (IC_50_ = 2.7 ± 0.3 µM) compared to its endogenous counterpart (156 ± 6.9 µM). Whilst these peptides may serve as good tools for characterising the enzyme *in vitro*, it may be difficult to develop them further as drug candidiates due to permeability and stability liabilities. 

Several classes of compounds were synthesized and evaluated for inhibitory activity against WNV helicase. However, as they showed differential inhibitory properties depending on whether an RNA or DNA substrate was used, it casts some doubts on the specificity of their inhibitory properties. For example, analogues of 1H-benzotriazole, 1H-benzimidazole, as well as AICAR (4-carbamoyl-5-(4,6-diamino-2,5-dihydro-1,3,5-triazin-2-yl)imidazole-1-beta-D-ribofuranoside) exerted good HCV and WNV helicase inhibitory activity when DNA was used as substrate. However, the activity was strongly decreased or even disappeared when RNA was used as a substrate [102,103]. Furthermore, a diverse library screen resulted in 5,6-dichloro-1-(beta-d-ribofuranosyl)benzotriazole (DRBT) which has good and selective inhibition of WNV helicase with an RNA substrate (IC_50_ ~ 0.3 µM), but much weaker with a DNA substrate (IC_50_ ~ 3 µM) [104]. This discrepancy in the inhibitory properties of these compounds suggests that the unwinding assays for WNV helicase could be further optimized. Finally, 5'-O-(4-fluorosulphonylbenzoyl)-esters of inosine exhibited low inhibitory activity against WNV helicase (IC_50_ = 70 µM with UTP substrate), but may not be specific as it was comparatively active against HCV polymerase (IC_50_ = 80 µM) [105]. 

A series of ring-expanded (“fat”) nucleoside analogues (RENs) containing the 6-aminoimidazo[4,5-e][1,3]diazepine-4,8-dione ring system with long C(12), C(14), or C(18) side-chains at position 6 were all found to have excellent profiles of activity and selectivity toward the viral versus cellular enzymes, with IC_50_ ranging between 1–10 µM for WNV helicase. One ring-expanded heterocycle analogue, which contains aralkyl substitution at position 1 (compound 39), was equally potent but somewhat less selective; whereas compound 36, which is an alpha-anomeric counterpart of 30, exhibited potent and selective inhibition of WNV (IC_50_ 1–3 µM). None of these compounds showed activity against the viral NTPase even up to 500 µM [106]. Likewise, RENs containing imidazo[4,5-e][1,3]diazepine ring system (compounds 14 and 15) and imidazo[4,5-e][1,2,4]triazepine ring systems (compound 30c) gave single-digit micromolar actvities against WNV helicase in a DNA or RNA unwinding assay [107]. The triphosphate forms of some of these compounds also inhibited WNV NTPase activity. Selectivity was also obtained against HCV, JEV, or human helicases. However, none of these compounds described in the literature, except for ivermectin [97] and imidazo[4,5-d]pyridazine nucleosides [108], possessed anti-WNV activity in cell culture. Even so, it was not proven that the inhibition of virus replication by these nucleoside analogs was due to direct effect on WNV helicase activity. The ability to generate resistant viruses that mapped to the helicase protein sequence would have provided strong evidence for on-target inhibition of these compounds.

### 3.4. NS4B

WNV NS4B is a small hydrophobic nonstructural protein that is hypothesized to participate in viral replication and evasion of host innate immune defenses [109]. Mutations in NS4B affect viral RNA replication [110,111,112], possibly through its interaction with NS3 helicase [113]. Several specific DENV-NS4B inhibitors have been identified through screening with whole-virus cell based assays [113,114], but thus far only one inhibitor has been reported for WNV. Lycorine was found to reduce viral titers of WNV, DENV-1 and -2, and YFV by 10^2^- to 10^4^-fold when tested at 1.2 µM concentration [115]. It exerted its antiviral activity mainly through suppression of viral RNA replication. WNV resistant to lycorine possessed a single amino acid substitution in V9M in the viral 2 K peptide (spanning the endoplasmic reticulum membrane between NS4A and NS4B proteins); this mutation increased viral RNA replication. Besides WNV and DENV, screening efforts also identified NS4B inhibitors that are selective for YFV [116].

### 3.5. NS5

**Figure 2:**
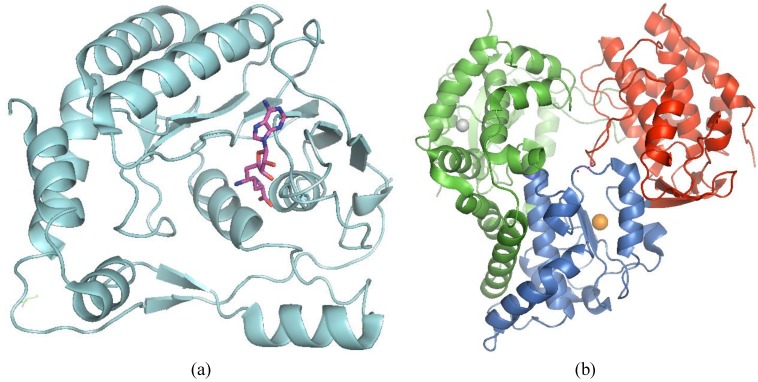
Crystal structures of West Nile Virus (WNV) NS5 MTase and RdRp domains depicted in cartoon representation. (a) WNV MTase (light blue) in complex with sinefugin (pink in stick representation; PDB code 3LKZ). Sinefungin inhibits Flavivirus MTase N7 and 2’-*O* activities by competitively binding to the enzyme catalytic pocket [121,128]; (**b**) WNV NS5 RdRp domain (blue) bound with Zn (grey sphere) and Mg^2+^ (orange sphere) coordinated by Asp536 and 669 (not labeled) [139,143]. Thumb, fingers, and palm subdomains are indicated in red, green and blue (PDB code 2HFZ).

NS5 is the most conserved protein amongst members of the genus *Flavivirus* and comprises the *N*-terminal MTase/GTase and the *C*-terminal RdRp. Separate crystal structures for several flavivirus MTase domains and RdRp domains have been solved and show very high structural homology (for reviews see [117,118,119,120]; Figure 2). Whilst both the capping and RdRp activities of NS5 are genetically validated to be essential for viral replication, only the RdRp activity can be considered as chemically validated, due to development of marketed drugs against viral polymerases such as for HIV-1 and HBV. Several polymerase inhibitors are currently in late phase clinical testing against the related HCV. Whilst majority of RdRp inhibitors are nucleoside analogs acting through chain termination of viral RNA replication, non-nucleoside inhibitors are also prevalent. It remains to be seen if the flavivirus MTase can also be successfully targeted, since the core domains of MTases are evolutionarily well conserved. Designing flavivirus selective inhibitors which do not inhibit host MTases, i.e., RNA MTase, DNA MTase, protein MTase, or the SAM-binding protein, is potentially challenging. On the other hand, it is envisaged that the RdRp would pose fewer selectivity issues as there are no structurally homologous enzymes in the host cell.

#### 3.5.1. MTase and GTase

WNV MTase performs sequential N-7 and 2'-*O* methylation of the viral RNA to generate a type-1 cap structure (reviewed in [121]). WNV defective in N-7 methylation is non-replicative; whilst WNV defective in 2'-*O* methylation is attenuated and can protect mice from subsequent wild-type WNV challenge [122]. 2'-*O* cap methylation also functions to subvert innate host antiviral response through modulation of the antiviral effects of a class of IFN-stimulated protein, the IFN-induced proteins with tetratricopeptide repeats (IFIT; [123]). This enzyme can further perform internal methylation of adenosines in the viral RNA genome at the ribose 2’-OH position [124]. Although binding of GTP and its analogues to the “GTP pocket” of flavivirus MTase domain has been shown for several different members of the family (reviewed in [117,118,119,120]), only two groups observed its GTase activity. Using radio-labeled GTP, they reported *in vitro* covalent attachment of GMP to Wesselsbron, DENV, YFV, and WNV MTase [125,126]. It has not been shown if such GMP-MTase covalent linkage is specific for GTP (i.e., if ATP, CTP or UTP has a similar activity). More work is required to definitively demonstrate the GTase activity of NS5. Collectively, these findings suggest that the dual N-7, 2'-*O* MTase/GTase is a potential target for flavivirus therapy. 

The wealth of structural information for NS5 MTase and RdRp domains has provided a solid foundation for structure-guided antiviral approaches. An extensive virtual screening campaign was performed by docking with more than 5 million commercial compounds into both the GTP-binding pocket and the active site of DENV MTase. Unfortunately, this exercise failed to yield any specific hits [127]. Taking advantage of a unique pocket located above the SAM-binding site of flavivirus MTase, a series of SAH analogs were rationally designed to bind into this site ([128,129]; Figure 2). Whilst the inhibitors were intended to target the DENV MTase, cross-reactivity to WNV MTase was observed [129]. Interestingly, the SAR appears to differ between the WNV and DENV MTases. WNV 2’-*O* MTase appears to be more susceptible to inhibition than the DENV MTase. This suggests that it may be possible to generate DENV- or WNV-specific inhibitors via this flavivirus MTase-conserved hydrophobic cavity. The challenge with this approach is the need to overcome the zwitterion nature of the SAH molecules to allow the compounds to entry the cells.

Using a GTP displacement fluorescent polarization assay with the YFV NS5 MTase domain, Geiss and colleagues performed a screen with a small library of 46,323 compounds. Six compounds were found to compete with GTP for bindng to both YFV and DENV MTase and also inhibited the DENV GTase activity [130]. The most potent compounds gave IC_50_ values of 5–8 µM. A second screening exercise was conducted with 235,456 compounds against the DENV NS5 MTase domain and identified a family of compounds, with a thioxothiazolidin core, that bound to both DENV and YFV MTases and inhibited DENV GTase [131]. One particular analog, (E)-{3-[5-(4-tert-butylbenzylidene)-4-oxo-2-thioxo-1,3-thiazolidin-3-yl]propanoic acid} (BG-323), possessed antiviral activity in DENV (EC_50_ 30.8 µM) cell culture with low toxicity (CC_50_ 184 µM). This compound also inhibited WNV (Kunjin) replication in cells, with up to 3 logs reduction in viral titre observed with 100 µM of BG-323. However, it is unclear if the cellular inhibition is directly due to effect on the viral MTase/GTase. 

#### 3.5.2. RdRp

Viral polymerase inhibitors can be classified into two broad categories: Nucleoside/nucleotide analog inhibitors (NIs) and non-nucleoside inhibitors (NNIs). NIs, when converted to its corresponding triphosphate, can compete with natural NTP substrates and be integrated into the growing chain of viral genome, blocking subsequent NTP incorporation. Incorporation of the inhibitors into the growing RNA template can also cause further mis-incorporations, inducing mutational error catastrophe, which leads to non-viable viral RNA templates. NNIs usually bind to allosteric sites in the polymerase and either “lock” the enzyme into an inactive form or prevent conformational changes required to initiate and/or elongate a new RNA product. 

NIs offer several advantages over NNIs. They target active sites, which are often conserved, and have a higher barrier of resistance [132]. Moreover, they often retain equivalent potency against different viral serotypes/genotypes and sometimes also work across related viruses. For example, a broad spectrum NI 5-aza-7-deazaguanosine was reported to inhibit DENV2, YFV, WNV, BVDV (bovine viral diarrhea virus), and Banzi virus at a single-digit micromolar concentration [133]. Cross-reactivity of nucleoside analogs to DENV1-4 and HCV has been reported by several groups [134,135,136,137]. Several NIs that are active against DENV also inhibit WNV. 7-deaza-2’-C-methyl-adenosine has EC_50_ of 4 and 15 µM against WNV and DENV1, respectively [138]. Rather unfortunately, no information is available on *in vivo* efficacy and toxicity. Two NIs have been profiled *in vivo*. NITD-008 (beta-D-2’-ethynyl-7-deaza-adenosine triphosphate) and NITD203 (3’,5’-*O*-diisobutyryl-2’-*C*-acetylene-7-deaza-7-carbamoyladenosine) have EC_50_s of <1 µM for all four serotypes of DENV and EC_50_ of ~5 µM for WNV. Both NIs could not reach a satisfactory no-observable adverse-effect level (NOAEL) in 2-week *in vivo* toxicity studies in rats and dogs [134,135]. These findings underline the fact that toxicity of nucleoside analogs is unpredictable and is one of the challenges related to this class of inhibitors. Nevertheless, based on the experiences of other infectious diseases (such as herpesviruses, HIV-1, HBV, and HCV), NIs remain the most promising class of compounds to succeed in the clinics. 

To date, there are no reported investigations of NNIs to WNV polymerase via diverse library screening or virtual screening. Unfortunately, researchers have not taken advantage of the relatively large amount of structural and biochemical information available on this enzyme [139,140,141]. One class of NNIs developed against DENV2 was not active against WNV polymerase [142]. This suggests that allosteric pockets may not be very well conserved in the two polymerases. Surface shape analysis of DENV3 and WNV RdRp crystal structures using *in silico* algorithms previously identified two cavities (A and B) common in the thumb domain of both proteins and additional three cavities unique to WNV ([143]; Figure 2). To determine if these cavities were potentially suitable for development of allosteric inhibitors, mutational analyses of conserved residues in these cavities was carried out in DENV2. Residues in cavity B, but not A, are critical for virus replication and mostly impaired NS5 polymerase activity *in vitro* [144]. Thus, cavity B could be used as a starting point for structure-based drug design or for virtual screening. The remaining three cavities in WNV RdRp, located in the thumb (cavity C and D) and in the finger subdomain (cavity E), have yet to be characterized. More recently, a co-crystal structure of DENV3 RdRp in complex with a small molecular weight inhibitor was reported [145]. It remains to be seen if this compound could be exploited as a starting point for designing inhibitors of WNV RdRp.

## 4. Inhibitors of Host Targets

“Hits” identified from the phenotypic cell-based screening could also inhibit host targets. The number of host factors required for a productive viral infection cycle is most probably higher than the number of virally encoded proteins (10 viral proteins in the case of WNV). Consequently, the probability of identifying inhibitors of host targets in cell-based assays would be greater than that for viral targets. The approach to target host proteins offers the advantage of a significantly higher barrier to spontaneous viral escape from inhibition, compared to direct antiviral agents, since viral mutations are less able to compensate for the loss of an essential host cofactor. Furthermore, since cellular replication of related viruses may involve similar host cell pathways, it may be possible to treat several viral indications with the same class of chemical compounds. For example, screening campaigns with DENV cell-based assays have uncovered compounds that also work on WNV (see below). The drawback of targeting host factors is the higher potential of undesirable drug-induced side effects, as these factors are often essential for cell survival or metabolism. For treatment of acute diseases, like dengue, this may be less of an issue compared to treatment of chronic diseases like HIV-1 or HCV [146]. For neurotropic flaviviruses like WNV, most symptomatic infections are associated with neuroinvasive disease and infection of neurons [147,148]. Thus, an additional consideration is that the compound must be able to cross the blood-brain barrier to be effective as a therapeutic agent.

### 4.1. Inhibitors of Viral Replication and Translation

Screening of the Novartis compound library with DENV induced cytopathic (CPE) assay led to the identification of a class of compounds with a benzomorphane core, which displayed a broad spectrum of anti-flavivirus activity [149]. It reduced the titer of DENV2, YFV, and WNV with EC_50_ of about 1, 4.9, and 4.5 µM respectively. The mode-of-action analysis indicated that the compounds inhibit protein translation in a viral RNA sequence-independent manner, but the exact protein that was affected by the compound was not identified. When tested in a dengue mouse viremia model, the most potent compound, NITD-451, reduced peak viremia by 40% at a low dose (25 mg/kg); but the compound resulted in adverse effects at higher doses. It would be interesting to determine the efficacy of this class of compounds in a WNV murine model and if the SAR established for DENV would track that for WNV.

A screening exercise using luciferase expressing WNV subgenomic replicon discovered one class of potent inhibitors that target viral translation [150]. Compound AP30451 exhibited an excellent EC_50_ of 60 nM and an SI of 533 in the WNV replicon assay; furthermore, the compound was active in neurons at a dose that did not cause significant cell toxicity. This compound also inhibited replication of DENV and YFV but not HCV replicons. Unfortunately, no further data is available on its further development.

Minocycline inhibited WNV replication and WNV-induced apoptosis in different human CNS-derived cell types with no evidence of cytotoxicity [151]. Minocycline acted through suppression of virus-induced activation of c-Jun *N*-terminal kinase (JNK) and its target c-jun. 

Cyclophilins, a family of cellular peptidyl-prolyl isomerases (PPIases), play a role in flavivirus replication [152]. Cyclosporine (an 11-amino-acid cyclic peptide known to block the PPIase activity of Cyclophilin A) inhibits WNV and DENV replication in cell culture at nontoxic concentrations. Time-of-addition and transient replicon results indicated that Cs inhibits flavivirus at the step of viral RNA synthesis. Biochemical analysis showed that Cyclosporine directly blocks the interaction between Cyclophilin A and WNV NS5 protein [152]. The results suggest that host Cyclophilin A is a component of flavivirus replication complex. The inhibitors of Cyclophilin currently in clinical development for HCV could potentially be repurposed for WNV and other flaviviruses.

### 4.2. Inhibitors of Host Pyrimidine or Purine Biosynthesis

Viral replication relies on the host to supply nucleosides. Host enzymes involved in nucleoside biosynthesis are potential targets for antiviral development. Ribavirin and mycophenolic acid inhibit replication of flaviviruses in part by suppressing inosine monophosphate dehydrogenase (IMPDH) enzyme activity, leading to depletion of the intracellular GTP pool [153,154,155]. Interestingly, the antiviral effects of both these compounds are augmented by induction of interferon-stimulated genes (ISGs) [156,157]. In addition, 6-azauridine (acts by blocking the conversion of orotic acid into UMP) has single digit micormolar inhibition against WNV. The utility of the latter two compounds is hampered by the cytostatic effects [158]. 

Recently, two classes of compounds were found to inhibit flavivirus replication by suppressing host pyrimidine biosynthesis. Firstly, NITD-982, a compound with an isoxazole-pyrazole core was identified from a CPE (cytopathic effect)-based HTS campaign [159]. It displayed nanomolar potency against four different RNA virus families, including *Flaviviridae*, *Paramyxoviridae*, *Orthomyxoviridae*, and *Retroviridae*. The compound was shown to inhibit the enzymatic activity of recombinant DHODH and to directly bind the DHODH protein. Inhibition of DHODH activity by NITD-982 resulted in depletion of intracellular pyrimidine pools, leading to the suppression of viral RNA synthesis. The second class of compounds, brequinar, also inhibited DHODH and could inhibit flaviviruses (DENV, WNV, YFV, and Powassan virus), alphavirus (Western equine encephalitis virus), and rhabdovirus (VSV) [160]. Supplementing the culture medium with pyrimidines reversed antiviral activities of both classes of compounds. Thus, the *in vitro* efficacy did not translate into *in vivo* efficacy and could be attributed to the uridine uptake from diets that replenish and maintain a high concentration of pyrimidine in plasma, which counteracted the compound-mediated inhibition of viral replication.

### 4.3. Inhibitors to Virus Assembly and Maturation

Celgosivir (butyl-castanospermine), is an oral prodrug of the natural product castanospermine, inhibits alpha-glucosidase I, an enzyme that plays a critical role in viral maturation by initiating the processing of the N-linked oligosaccharides of viral E and NS1 glycoproteins. It was tested in a phase II clinical trials in combination with peglated IFN and ribavirin for treatment of HCV infection; addition of celgosivir did not show any benefit when compared with the standard IFN/ribavirin regime [161]. Several studies have indicated that castanospermine and celgosivir also inhibits DENV replication in cell cultures and mouse models [162,163,164]. Celgosivir has been recently tested in dengue patients; the efficacy has not been published. Notably, celgosivir did not show any protective effect on WNV-infected cells or mice [162]. Imino sugars, such as N-butyl-deoxynojirimycin (DNJ) and N-nonyl-deoxynojirimycin (NNDNJ), inhibit both alpha-glucosidase I and II. Derivatives of DNJ, such as N-pentyl-(1-hydroxycyclohexyl)-DNJ (OSL-95II), PBDNJ0801, PBDNJ0803, and PBDNJ0804, had micromolar antiviral activity against BVDV, WNV, DENV, and HBV without observable cytotoxic effects [165,166]. 

Several host proteins have been reported to be involved in virus assembly and maturation. These may potentially be explored as host target to inhibit virus particle formation and dissemination. The host nucleolar helicase DDX56/NOH61 interacts with WNV capsid protein and is essential for assembly of infectious WNV virions [167,168]. The src family kinase (SFK), c-Yes, has also been implicated to play a role in transit of WNV particles through the secretory pathway. Treatment of WNV-infected cells with the SFK inhibitor PP2 reduced the E protein glycosidation, leading to accumulation of virions in the ER compartment [169]. Recent advances in development of peptidomimetic furin inhibitors yielded in compounds with picomolar activity and enhanced stability in cell culture. They inhibited the hemagglutinin cleavage and viral propagation of a highly pathogenic avian H7N1 influenza virus strain [170]. Inhibitors of furin or furin-like proprotein convertases could represent promising antiviral drug candidates for infectious diseases such as WNV.

## 5. Discussion and Perspectives

Compared with HCV or DENV, the effort for WNV drug discovery is much lower. This may be due to the perception that there is not an urgent need for antivirals against this disease. Most human infections with WNV are asymptomatic, with about 20% of individuals developing flulike symptoms with high fever. Only about 1% of cases develop severe neuroinvasive disease (encephalitis, meningitis or flaccid paralysis). Infection with WNV is mostly transient and leaves a life-long immunity although persistent infections have been demonstrated occasionally. With time, it is expected that WNV seroprevalence will increase both in the human and bird populations, resulting in increasing immunity and a reduction in outbreaks [171]. Nevertheless, given the mutability of the virus, in particular for enhanced neuroinvasiveness, it is possible that new waves of WNV outbreak may take place from newly emerging virulent isolates. Under these circumstances, antiviral remains the best hope for intervention. Due to the homology among different flaviviruses, antivirals developed for DENV may potentially be useful for WNV and other flaviviruses. In both laboratory and clinical settings, the genetic barrier to drug resistance varies depending on the classes of DAAs. The phenotypes of compound-resistant mutant viruses cultured under laboratory settings also depends on the selection protocol used, and do not necessarily correlate with mutations observed in drug-treated patients [132,172]. In general, nucleoside analogues have a much higher barrier to resistance than protease and NIs. Any treatment regime envisaged for WNV disease would benefit with the incorporation of a NI.

## Abbreviations

DENV: dengue virus
WNV: West Nile virus
YFV: yellow fever virus
TBEV: tick-borne encephalitis virus
HCV: hepatitis C virus
WEEV: Western equine encephalitis virus
VSV: vesicular stomatitis virus
HBV: hepatitis B virus
HIV: human immunodeficiency virus
DAA: direct antiviral agent
GTase: guanylyltransferase
PI: protease inhibitors
HTS: high-throughput screening
MTase: methyltransferase
RdRp: RNA-dependent RNA polymerase
NI: nucleoside analog inhibitor
NNI: non-nucleoside inhibitor
TI: therapeutic index
SAR: structure-activity relationship
ER: endoplasmic reticulum
